# Physiological and gene expression analysis of *Hevea brasiliensis* under drought stress

**DOI:** 10.1371/journal.pone.0338177

**Published:** 2025-12-30

**Authors:** Mostafa Mohammadi Nodehi, Mohtaram Mahmoudieh, Alireza Taleei, Valiollah Mohammadi, Mohammad Mahbubur Rahman, Meisam Zargar, Mohammad Reza Naghavi

**Affiliations:** 1 Division of Biotechnology, Department of Agronomy and Plant Breeding, College of Agricultural and Natural Resources, University of Tehran, Karaj, Iran; 2 Silviculture Genetics Division, Bangladesh Forest Research Institute, Chittagong, Bangladesh; 3 Department of Agrobiotechnology, Agrarian Technological Institute, RUDN University, Moscow, Russia; Nuclear Science and Technology Research Institute, IRAN, ISLAMIC REPUBLIC OF

## Abstract

Natural rubber, derived mainly from the *Hevea brasiliensis* tree, is a highly valuable biopolymer. This study examined the effects of drought stress on rubber seedlings, focusing on their physiological responses and gene expression under three irrigation conditions: well-watered (control), mild drought, and severe drought. Results indicated that as drought severity increased, the relative water content in the leaves decreased. The level of proline was significantly higher under severe drought but decreased during mild drought stress. Malondialdehyde levels increased in leaves under drought stress, while antioxidant enzymes varied: ascorbate peroxidase and catalase activity increased under mild drought stress. The guaiacol peroxidase (GPX) activity rose under drought condition, indicating adaptive oxidative and osmotic responses. Gene expression analysis demonstrated significant down-regulation of the rubber biosynthesis gene 3-hydroxy-3-methylglutaryl-CoA reductase (*HMGR*) under drought conditions, indicating a reduction in rubber production. In contrast, the expression of cis-prenyltransferase was up-regulated, suggesting a compensatory mechanism to maintain rubber synthesis despite a shortage of precursors. HMG-CoA synthase significantly decreased under severe drought stress, whereas transferase activator exhibited non-significant changes during drought conditions. Additionally, an inverse relationship was identified between *HMGR* expression and GPX activity, suggesting that increased levels of reactive oxygen species during drought stress may inhibit antioxidant responses, ultimately leading to the down-regulation of *HMGR*. Drought stress suppresses *HMGR* expression, reducing rubber yield. Preventing the downregulation of this gene under drought conditions could be a key focus for future research. These findings enhance our understanding of the molecular mechanisms of drought adaptation in rubber seedlings and provide insights for breeding resilient genotypes.

## Introduction

Natural rubber (NR), mainly composed of cis-1,4-polyisoprene, is a crucial biopolymer used in industries like automotive, medical, and manufacturing [1]. Although over 2,500 plant species produce rubber [2], only *Hevea brasiliensis* is commercially viable, supplying about 89% of global natural rubber [1,3,4]. In rubber seedlings, rubber biosynthesis occurs in specialized cells called articulated laticifers, primarily located in the phloem of stems, with some in leaves and branches [5–7]. These cells generate latex, a cytoplasmic suspension rich in rubber particles and enzymes for the isoprenoid biosynthesis pathway [8,9].

The rubber synthesis pathway is energy intensive and particularly sensitive to ecological stress conditions [10]. Due to increasing climatic instability, understanding responses of rubber seedlings to environmental stress is essential for establishing long-term productivity. Drought stress is a major global constraint that increases reactive oxygen species (ROS) and inhibits respiration, partially due to reduced antioxidants [11]. Enzymes such as ascorbate peroxidase (APX), catalase (CAT), and guaiacol peroxidase (GPX) play key roles in detoxifying ROS and protecting plants from oxidative damage [12]. However, excessive ROS can hinder crop productivity by inducing physiological disruptions such as membrane lipid peroxidation, protein misfolding, and metabolite degradation [13,14]. This study aims to investigate the effects of drought on growth traits, antioxidant enzyme activities, osmolyte accumulation, and expression of four key genes (*HMGR*, *HMGS*, *RTA*, and *CPT*) involved in rubber biosynthesis [10,15]. Elucidating these factors will enhance our understanding of the mechanisms behind drought tolerance in rubber seedlings. This knowledge will support future breeding and cultivation efforts under drought-prone conditions.

## Materials and methods

### Plant material, experimental design, and growth conditions

**Fig 1:**
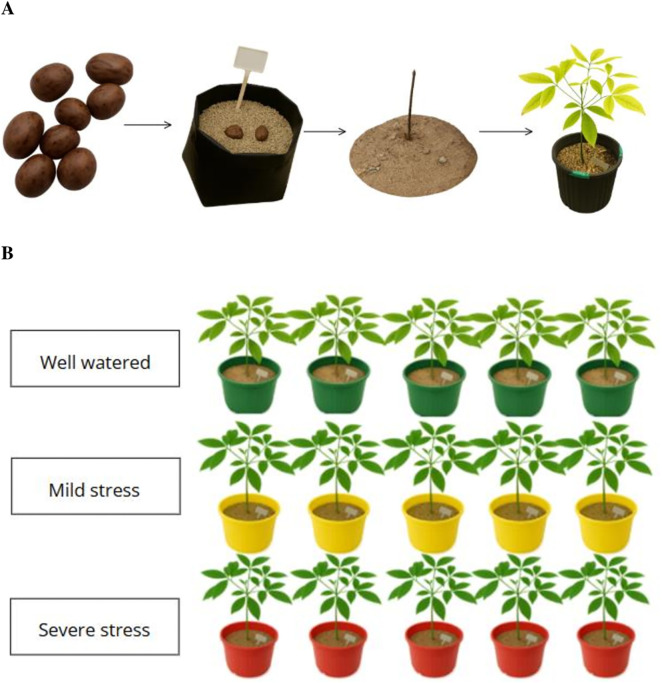
The experimental design used for drought stress treatments of *Hevea brasiliensis* seedlings. Seed germination and *Hevea brasiliensis* seedling production **(A)**, drought stress treatments were conducted using three irrigation regimes (well-watered, mild drought, and severe drought) **(B)**. Five biological replicates (n = 5) were used for each treatment group (each pot serving as a separate replicate).

Seeds of the rubber tree (clone RRIB-600) were soaked in water for 24 hours at room temperature and then sown in black polyethylene nursery bags filled with washed river sand. The seeds were lightly covered with sand and kept in a greenhouse at 28–30 °C with humidity above 60%, with daily watering to maintain soil moisture. Seed germination was observed between 13 and 17 days after sowing. Eight-week-old seedlings, approximately 25 cm long, were transplanted into plastic pots containing a loamy-sandy textured substrate. The plants were grown in a controlled greenhouse environment with temperatures between 28 and 30 °C, relative humidity levels of 60–70%, and a 12-hour light/dark photoperiod. Six-month-old seedlings (15 seedlings) were selected for morphological assessment, with five biological replicates (n = 5) per treatment group (each pot representing a separate biological replicate). A summary of the experimental design is illustrated in Fig 1.

### Drought stress treatments and morphological assessment

Six-month-old rubber seedlings were subjected to drought treatments for 42 days using fixed daily irrigation volumes methods as previously described [16,17]. In brief, they received 50 mL/day for the well-watered control, 20 mL/day for mild drought stress, and 2 mL/day for severe stress. Irrigation was performed manually every 2–3 days to ensure consistent water delivery.

Morphological evaluations were conducted at the beginning and end of the treatment period, measuring plant height and stem diameter. At the end of treatments, fully expanded leaves were harvested, flash-frozen in liquid nitrogen, and stored at –80 °C for biochemical and molecular analyses.

### Physiological assays

#### Relative water content (RWC).

RWC was estimated as described previously [18,19]. Briefly, the fresh weight (FW) was measured immediately after sampling from each plant. The leaves were then floated in distilled water for 6 hours, and the turgid weight (TW) was recorded. Afterward, the leaves were oven-dried at 70°C for 48 hours, and the dry weight (DW) was determined. The RWC was calculated using the following formula [19]:


Rwc(%)=[(FW−DW)/(TW−DW)]×100

#### Proline quantification.

Free proline was extracted and quantified using the acid ninhydrin method [20,21]. Briefly, 0.5 g of frozen leaf tissue was homogenized in 10 mL of 3% sulfosalicylic acid and vortexed for 30 seconds. The mixture was then centrifuged (Sigma Qiagen Model 4–16KS, Germany) at 22,500 xg for 15 minutes at 4°C. The supernatant was filtered through filter paper. 2 mL of the filtrate were mixed with 2 mL of acid ninhydrin and 2 mL of glacial acetic acid, then incubated at 65°C for 1 hour and cooled on ice. The chromophore was extracted with 4 mL of toluene, and the absorbance of the upper phase (colored solution) was read at 520 nm using a plate reader (EON Biotek, Highland Park, Winooski, Vermont, USA). Concentrations were determined from a standard curve of pure proline, expressed as micrograms per gram of dry weight.

#### Malondialdehyde (MDA) content.

MDA, a marker of lipid peroxidation, was measured using the thiobarbituric acid (TBA) assay [22]. 1 g of fresh leaf tissue was homogenized in 5 mL of 0.1% trichloroacetic acid (TCA), followed by centrifugation at 10,000 xg for 5 minutes at room temperature. 1 mL of the supernatant was mixed with 4 mL of 20% TCA containing 0.5% TBA and incubated at 95°C for 30 minutes. After cooling on ice and re-centrifugation, absorbance was measured at 532 nm and 600 nm. The MDA content was calculated using an extinction coefficient of 155 mM ⁻ ¹cm ⁻ ¹.

### Antioxidant enzyme assays

For enzyme assays, 0.2 g of leaf tissue was homogenized in 1 mL of extraction buffer containing 1 M Tris-HCl (pH 6.8) and 2% polyvinylpyrrolidone (PVP). Samples were vortexed for 30 seconds and centrifuged at 22,500 xg for 30 minutes at 4°C [23]. Supernatants were collected and stored at –20°C until analysis.

Catalase (CAT) activity was assayed by measuring the decomposition of 50 mM H₂O₂ in 50 mM phosphate buffer (pH 7.0). An aliquot (10 μL) of enzyme extract was mixed with reaction buffer at a dilution ratio of 1:200, and the decrease in absorbance was recorded at 240 nm [24,25].

To assess ascorbate peroxidase (APX) activity a reaction mixture containing 50 mM phosphate buffer (pH 7.0), 0.5 mM ascorbate, and 1 mM H₂O₂ was prepared [26]. The APX activity was measured by monitoring the decrease in absorbance at 290 nm for 2 minutes. The activity was expressed as ΔA 290 mg ^−1^ protein min ^−1^.

Guaiacol Peroxidase (GPX) activity was determined by measuring the formation of tetra guaiacol, which absorbs at 470 nm [27]. Equal volumes of 10 mM phosphate buffer, guaiacol (10 mM), and H₂O₂ were mixed. The enzyme extract was added to the mixture at a dilution ratio of 1:200. The increase in absorbance was recorded over 2 minutes at 470 nm.

### Quantitative real-time PCR analysis

Total RNA was extracted from approximately 100 mg of frozen leaf tissue using the DenaZist Total RNA kit (DenaZist, Iran), following the manufacturer’s instructions for non-column-based RNA isolation. The RNA was quantified with a Nanodrop spectrophotometer and assessed using 1% agarose gel electrophoresis. DNase treatment was then performed using the DNase I enzyme kit (Thermo Fisher Scientific, USA) to eliminate any residual genomic DNA contamination. Reverse transcription was conducted using a Supra cDNA Synthesis Kit (Pars Tous, Iran) to convert RNA into complementary DNA (cDNA). The following combinations of forward and reverse primers were used: for the quantitative real-time PCR for *CPT* 5′-TGTCATAGCTTCTCGCCCAA-3′/5′-ATGGTGACGTACTTAACTCCGAT-3′; for *HMGR* 5′-CCGTTTTCAACAAATCAAGCCGAT-3′/5′-ACCATGTTCATCCCCATTGCATC-3′; for *HMGS* 5′-CCATAGGACTCGCACAAGATTGC-3′/5′-GATTACCGTTTCACTCCCGACTTC-3′; and for *RTA* 5′-CGGACCCCAGAAGATTTATCGC-3′/5′-CTCTCCACCACAATTCCAAGATA-3′ [23]. The quantitative real-time PCR was performed using SYBR Green master mix (Pars Tous, Iran) in a Rotor-Gene 6000 series system (QIAGEN’s real-time PCR system), following the manufacturer’s instructions. The PCR conditions included: 95°C for 15 minutes, followed by 40 cycles of 95°C for 15 seconds; 60–62°C (depending on the annealing temperature) for 20 seconds; and 72°C for 20 seconds.

### Statistical analysis

A factorial experiment based on a completely randomized design was conducted with 5 replicates. Data analysis was performed using one-way ANOVA with SAS software (version 9.4), and a *p*-value of 0.05 was used to determine statistical significance. The 2^-ΔΔCT^ method [28] was applied to analyze the relative expression levels of each selected gene with three technical replicates. Graphs were plotted using GraphPad Prism (version 9.0).

## Results

### Morphological and physiological responses to drought stress

**Fig 2:**
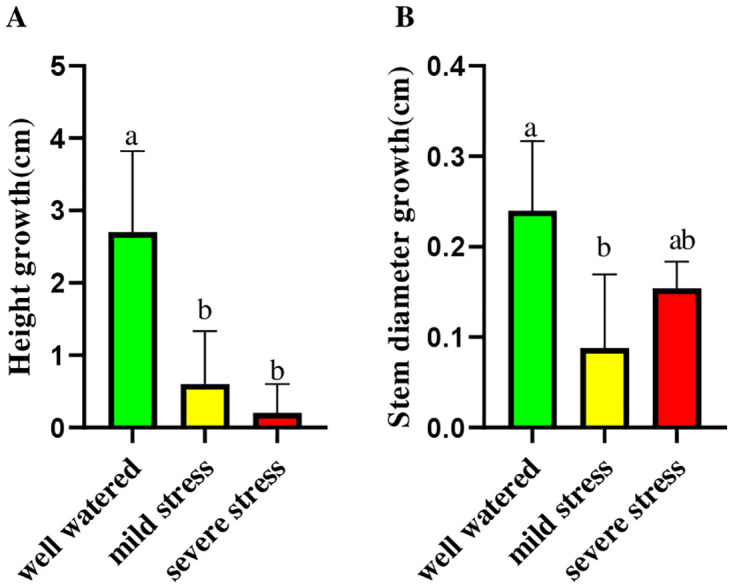
The effect of drought stress on the morphological traits of *Hevea brasiliensis* seedlings. The impact of drought stress on height growth **(A)**, and stem diameter growth **(B)**. The values are presented as mean ± standard error (SE) of 5 replications (n = 5). The values followed by different letters are significantly different (*p* < 0.05).

Drought stress significantly impacted all measured morphological traits of rubber seedlings. The results showed that plant height decreased under drought conditions. Well-watered (control) plants had an average growth of 2.7 ± 1.12 cm, while mild and severe drought conditions resulted in only 0.6 ± 0.73 cm and 0.2 ± 0.4 cm growth, respectively. Stem diameter increased under severe drought (0.154 ± 0.029 mm) compared to mild drought stress (0.088 ± 0.081 mm) and control (0.24 ± 0.076 mm) (Fig 2).

Physiological markers indicated water deficit stress and activation of defense mechanisms: Relative water content (RWC) was significantly reduced with increasing stress severity. Well-watered (Control) plants maintained high RWC (92.17 ± 2.10%), while mild and severe drought treatments resulted in 68.86 ± 1.16% and 53.62 ± 2.24%, respectively. Proline content, an osmo protectant [29], increased significantly under severe drought (0.057 ± 0.006 μg/g DW) compared to mild drought stress (0.0096 ± 0.0012) and control (0.026 ± 0.0034). Malondialdehyde (MDA) levels increased significantly (*p* value = 0.00026) under drought, from 40.02 ± 7.63 nmol/g FW in the control to 111.05 ± 22.73 and 217.87 ± 22.63 under mild and severe drought stress, respectively, indicating increased oxidative damage.

**Fig 3:**
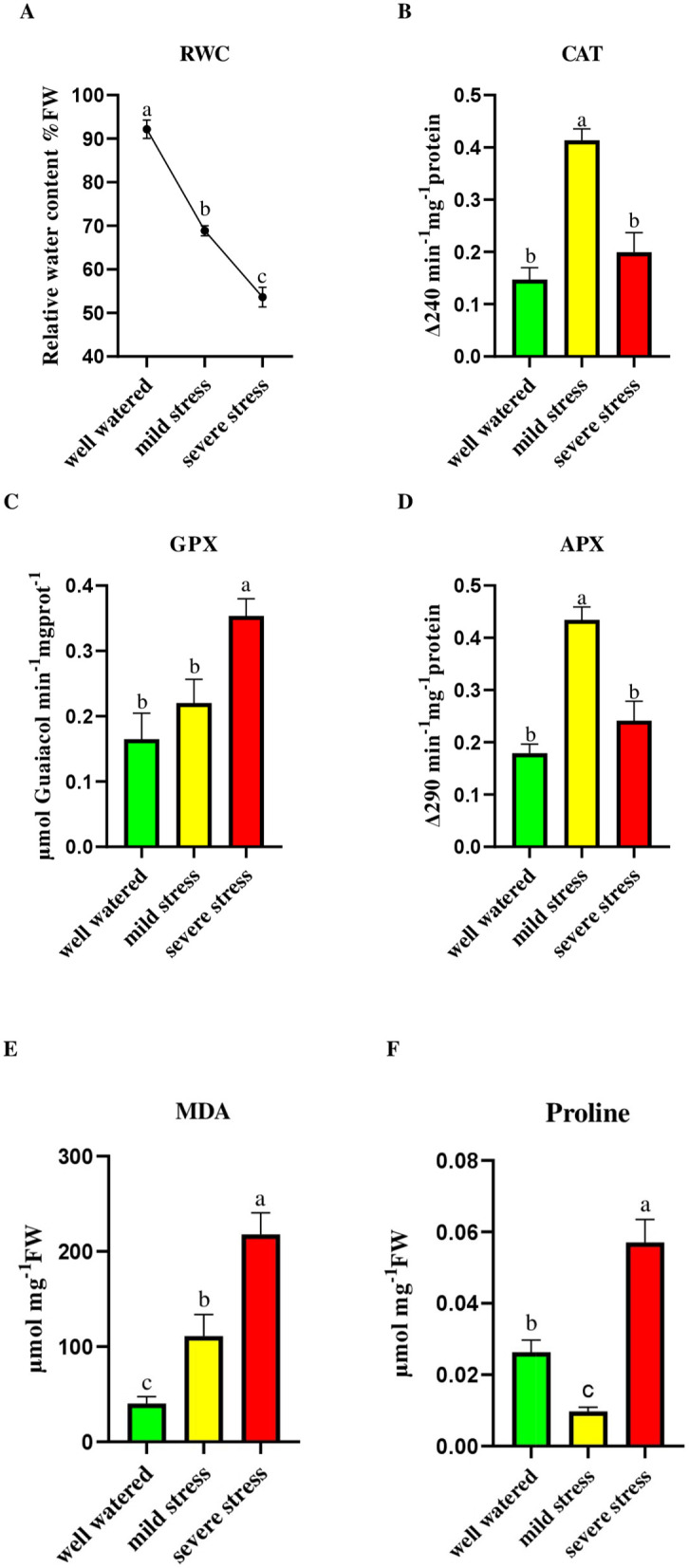
Effect of drought stress on physiological traits of *Hevea brasiliensis* seedlings. Relative water content **(A)**, catalase **(B)**, glutathione peroxidase **(C)**, ascorbate peroxidase **(D)**, malondialdehyde **(E)**, and proline **(F)**. Treatments are: well-watered (control), mild drought and severe drought. The values are presented as mean ± standard error (SE) of 5 replications (n = 5). The values followed by different letters are significantly different (*p* < 0.05).

Catalase (CAT) activity displayed a biphasic response: It increased under mild drought stress (0.41 ± 0.021 U/mg protein) compared to control (0.14 ± 0.022), but decreased under severe stress (0.19 ± 0.037). Ascorbate peroxidase (APX) activity was lowest in control conditions (0.18 ± 0.018), peaked during mild drought (0.43 ± 0.024), and then declined with severe drought (0.24 ± 0.037), indicating a temporary upregulation in response to mild drought stress followed by suppression under more intense conditions. Guaiacol peroxidase (GPX) steadily increased with drought severity, from 0.16 ± 0.039 in control to 0.22 ± 0.036 and 0.35 ± 0.026 under mild and severe drought stress, respectively, highlighting its essential role in long-term ROS detoxification (Fig 3).

### Expression of genes relevant to rubber biosynthesis

**Fig 4:**
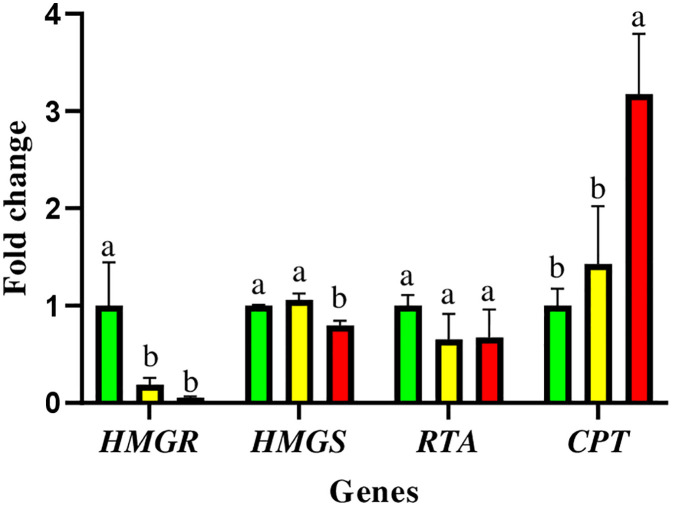
Quantitative real-time PCR analysis of rubber biosynthesis genes. Rubber biosynthesis genes are 3-hydroxy-3-methylglutaryl-CoA reductase (*HMGR*), HMG-CoA synthase (*HMGS*), rubber transferase activator (*RTA*), and Cis-prenyltransferase (*CPT*). The values are presented as mean ± standard error (SE) of the average of biological replicates (n = 3). The values followed by different letters are significantly different (*p* < 0.05).

The results showed that the expression patterns of genes involved in rubber biosynthesis were significantly altered by drought stress: 3-hydroxy-3-methylglutaryl-CoA reductase (*HMGR*) expression declined under stress compared to well-watered (control) plants. While it decreased to 0.19 ± 0.068 in mild drought stress and further to 0.055 ± 0.012 in severe stress, indicating repression of upstream isoprenoid biosynthesis. HMG-CoA synthase (*HMGS*) expression significantly decreased under severe drought stress (0.79 ± 0.051), while it showed a slight increase in response to mild drought stress (1.05 ± 0.064); however, this increase was not statistically significant. The expression of the rubber transferase activator (*RTA*), which activates cis-prenyltransferase [30], was downregulated from 1 in the control to 0.65 ± 0.26 and 0.67 ± 0.28 under mild and severe stress, respectively; however, these changes were not statistically significant. Cis-prenyltransferase (*CPT*), a crucial enzyme involved in polymer elongation [31], demonstrated a significant upregulation under severe stress condition (3.17 ± 0.62) (Fig 4).

## Discussion

This study demonstrates a closely coordinated and complex response of rubber seedlings to increasing drought stress, involving changes in morphology, function, and gene expression. The drought simulation method, which employed a controlled and gradual reduction in watering, successfully created distinct stress levels, as confirmed by significant differences in relative water content (RWC) among treatments (*p* < 0.05). Table 1 summarizes the ANOVA results, showing that drought treatments significantly influenced most measured traits and supporting further analysis.

**Table 1:** Analysis of variance (ANOVA) for morphological, physiological and gene expressions under different treatments.

Trait	Treatment		Error	
	Df	Ms (Between groups)	Df	Ms (within groups)
Height growth	2	9.02^*^	12	0.82
Stem diameter growth	2	0.03^*^	12	0.01
RWC	2	1131.09^*^	6	5.41
MDA	2	24042.25^*^	6	543.83
Proline	2	0.0017^*^	6	0.00002
CAT	2	0.06^*^	6	0.0017
APX	2	0.05^*^	6	0.0017
GPX	2	0.03^*^	6	0.0017
*HMGR*	2	0.78^*^	6	0.1
*HMGS*	2	0.05^*^	6	0.0033
*RTA*	2	0.08^ns^	6	0.08
*CPT*	2	4.05^*^	6	0.38

Df: degree of freedom, *p < 0.05, ns = not significant, Ms = mean square.

**Fig 5:**
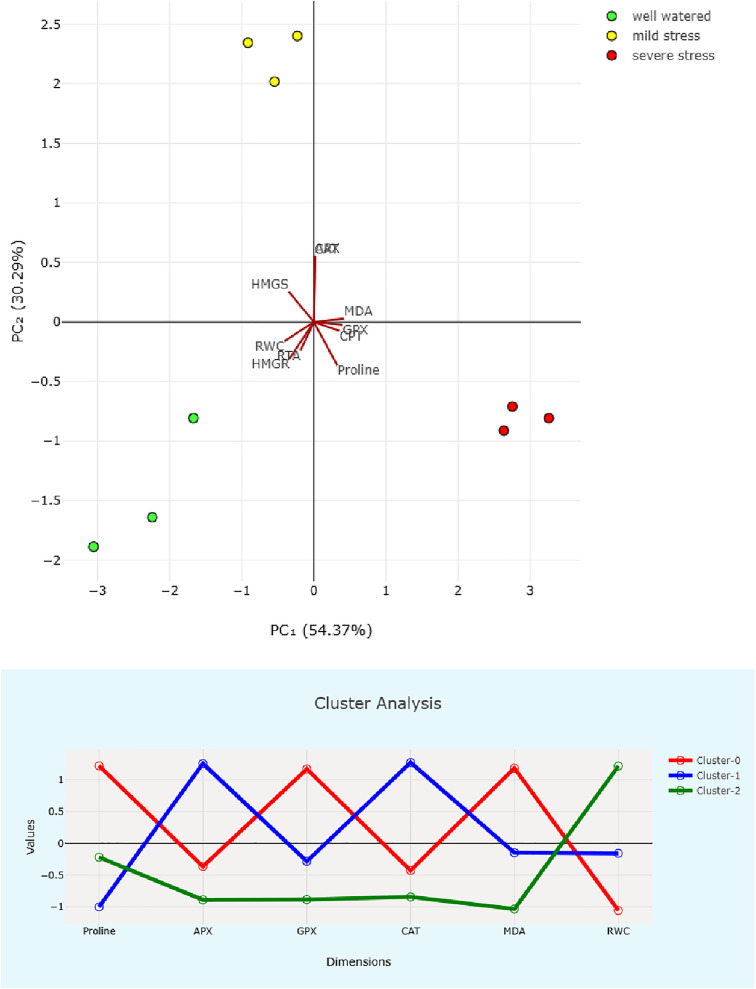
Principal component analysis and cluster analysis of physiological and molecular traits in response to drought stress. Principal Component Analysis (PCA) effectively differentiated the three treatment groups along PC1 and PC2. The traits are: relative water content (RWC), proline, ascorbate peroxidase (APX), glutathione peroxidase (GPX), catalase (CAT), malondialdehyde (MDA), 3-hydroxy-3-methylglutaryl-CoA reductase (*HMGR*), HMG-CoA synthase (*HMGS*), rubber transferase activator (*RTA*), and Cis-prenyltransferase (*CPT*).

Principal Component Analysis (PCA) effectively separates the three treatment groups along PC1 and PC2, which collectively accounted for 84.66% of the total variance. Notably, traits like RWC, *HMGR*, and *HMGS* were grouped with well-watered samples. In contrast, antioxidant enzymes such as CAT and APX were associated with mild drought stress, while proline, MDA, and *CPT* were linked to severe drought conditions. Previous studies shown that drought has a significant impact on the biochemical characteristics of fruits. The study by Ünal and Okatan, [32] highlights drought-induced shifts in phytochemical profiles, in which the contents of total phenolics and total anthocyanins in strawberry varieties were found to increase under drought stress conditions [32]. Cluster analysis of physiological traits further supports this transition, grouping traits into stress-dependent modules (Fig 5). The mild drought stress cluster is characterized by high antioxidant activity. Conversely, the severe stress cluster exhibits increased oxidative damage and osmolyte accumulation, along with suppression of energy-demanding biosynthetic genes like *HMGR*. These patterns suggest an adaptive tradeoff between ROS mitigation and biosynthetic energy use, possibly mediated by nicotinamide adenine dinucleotide phosphate (NADPH) availability.

**Fig 6:**
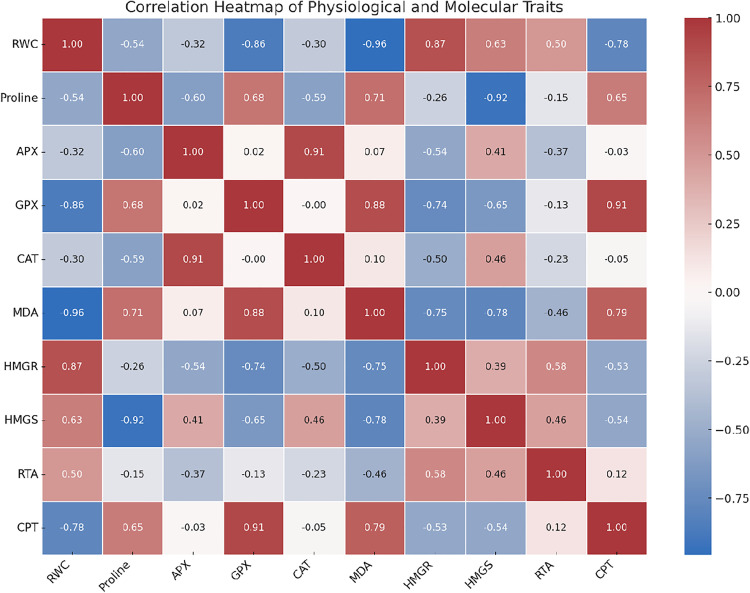
Heatmap analysis of physiological and molecular traits across different data sets. The distribution and intensity of measured data, including relative water content (RWC), proline, ascorbate peroxidase (APX), glutathione peroxidase (GPX), catalase (CAT), malondialdehyde (MDA), 3-hydroxy-3-methylglutaryl-CoA reductase (*HMGR*), HMG-CoA synthase (*HMGS*), rubber transferase activator (*RTA*) and Cis-prenyltransferase (*CPT*) across different treatments are shown.

A heatmap of the data presented in Fig 6 visually illustrates the distribution and intensity of the measured data across various treatments. The high-density areas represented in red and low-density areas in blue. This visual tool enhances comprehension and facilitates more effective analysis of the core information.

### Morphological adaptations: resource allocation under stress

The significant reduction in plant height under drought conditions indicates a strategic adjustment in resource allocation by rubber seedlings, prioritizing survival over vertical growth. By restricting shoot elongation, these plants likely reduce their transpirational surface area and metabolic demands, thereby conserving scarce water resources [33]. Although some studies suggested that increased drought intensity led to reduced stem diameter growth [34–36], this study revealed a different trend, showing that stem diameter growth slightly increased during severe drought compared to mild drought stress. This suggests an adaptive structural change, likely aimed at enhancing mechanical stability and increasing the plant’s capacity for water storage and axial transport through the reinforcement of vascular tissues [37]. The observed variation in biomass allocation under drought stress underscores a strategic shift in growth priorities, favoring structural reinforcement, such as an increase in stem diameter, over vertical elongation. This adjustment aligns with established patterns of resource allocation focused on survival in woody perennials facing water deficit conditions [38,39].

### Physiological responses: oxidative stress and osmotic adjustment

The progressive and statistically significant decline in relative water content (RWC) across different treatments confirmed the successful application of drought stress. It indicated increasing levels of physiological dehydration in rubber seedlings. This is consistent with the findings of Wang’s study, which demonstrated that RWC in leaves continuously decreased with increasing drought severity, leading to the observation of wilting leaves [40].

In current study, the mild drought treatment resulted in significantly lower proline accumulation compared to both the well-watered control and the severe drought group. Additionally, in the mild drought treatment, there was a notable increase in the activities of antioxidant enzymes, particularly CAT and APX. In contrast, severe drought caused a significant decline in CAT and APX activities, accompanied by a substantial increase in proline accumulation. This inverse pattern indicates that the defense strategy in *H*. *brasiliensis* differs based on stress severity and *H*. *brasiliensis* favors rapid enzymatic antioxidant responses over the biosynthesis of non-enzymatic osmolytes [29,41,42]. Under prolonged and intense oxidative stress, rubber trees may shift their defense strategies and adopt a protective strategy focused on osmolytes instead of relying on enzymatic detoxification [43,44]. Previous studies on *H*. *brasiliensis* have demonstrated that drought treatments significantly increase proline accumulation [45,46]. In Stevia, Khan et al., [47] reported that drought stress resulted in elevated levels of MDA, proline, APX, and CAT, accompanied by a reduction in plant height [47]. Similarly, Li and Tu, [48] observed that drought stress in Oxalis led to increased levels of H₂O₂, MDA, and CAT, along with decreases in both plant height and stem diameter [48]. These findings are consistent with the morphological and physiological changes observed in our study on rubber seedlings.

GPX activity showed a steady increase, indicating a more stable and long-term role in oxidative protection. The increase in GPX activity was associated with a rise in proline content. Additionally, lipid peroxidation, indicated by elevated MDA levels, significantly increased with drought severity, highlighting the accumulation of oxidative damage in plant tissues. Both mild and severe drought treatments caused statistically significant increases in MDA content compared to the control group, confirming that water deficit stress accelerates membrane lipid degradation [49,50]. This comprehensive understanding of stress responses in *H*. *brasiliensis* is valuable for future strategies for enhancing drought resilience in rubber cultivation.

### Molecular regulation of rubber biosynthesis under drought

The *HMGR* gene, which plays a crucial regulatory role in the mevalonate (MVA) pathway [51,52], was significantly downregulated under stress. During drought-induced oxidative stress, excessive accumulation of ROS leads to increased consumption of NADPH by antioxidant systems [53–55]. Given that the *HMGR* activity is significantly influenced by NADPH levels [56–58], its downregulating could be an adaptive mechanism in response to low NADPH availability, emphasizing the prioritization of ROS detoxification over the synthesis of isoprenoids.

**Fig 7:**
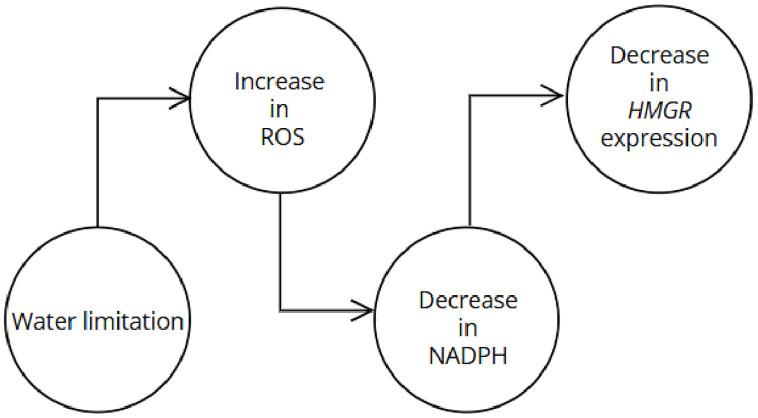
A schematic diagram representing the impact of drought stress on ROS, NADPH consumption, and *HMGR* gene expression. Under drought-induced oxidative stress, excessive accumulation of reactive oxygen species (ROS) results in increased nicotinamide adenine dinucleotide phosphate (NADPH) consumption by antioxidant systems, following a decrease in the expression of the 3-hydroxy-3-methylglutaryl-CoA reductase (*HMGR*) gene. This is due to activation of the SNF1-related protein kinase 1 (SnRK1), which suppresses anabolic gene expression [59,60].

Multiple linear regression analysis was conducted to understand the relationship between ROS enzyme activity and *HMGR* expression. This analysis revealed that *HMGR* expression had a negative association with GPX activity (β = –2.88, p = 0.0042). The regression model accounted for 69.4% of the variance in *HMGR* expression (adjusted R^2^ = 0.694), indicating that increased oxidative stress, particularly elevated GPX activity, is statistically associated with decreased *HMGR* transcription. Additionally, correlation analysis confirmed a negative correlation between *HMGR* gene expression and ROS enzyme activity. A schematic diagram of the impact of drought stress on ROS, NADPH consumption, and *HMGR* gene expression is presented in Fig 7.

The coordinated expression patterns of *HMGR*, *HMGS*, and *CPT* suggest a drought-induced shift in regulatory priorities within the rubber biosynthesis pathway of the rubber tree. Suppression of upstream genes (*HMGR* and *HMGS*) likely reflects limited precursor availability due to oxidative stress. At the same time, the strong upregulation of *CPT* indicates a compensatory mechanism to maintain rubber chain elongation, possibly decoupled from precursor synthesis. This inverse expression pattern highlights a potential cross-regulatory adaptation aimed at preserving rubber quality under drought stress. Although RTA transcript levels exhibited a downward trend under both mild and severe drought conditions, statistical analysis revealed no significant difference among treatments, suggesting that *RTA* may not be transcriptionally responsive to drought stress in rubber seedlings.

## Conclusion

This study provides valuable insights into the physiological and molecular responses of rubber seedlings (*H*. *brasiliensis*) to drought conditions. Our findings demonstrate that drought stress significantly impacts the relative water content, proline accumulation, and oxidative stress levels in rubber seedlings, highlighting the challenges of water scarcity. Changes in antioxidant enzyme activities, particularly ascorbate peroxidase, catalase, and guaiacol peroxidase, reveal the complexity of the seedlings’ adaptive mechanisms to oxidative stress. Gene expression analysis indicates critical regulatory shifts in key biosynthetic pathways under drought. The down-regulation of the *HMGR* gene suggests a direct impact on rubber production, while the up-regulation of the *CPT* gene indicates a compensatory mechanism to sustain rubber synthesis despite precursor limitations. The inverse relationship between *HMGR* expression and guaiacol peroxidase activity demonstrates the balance between reactive oxygen species and antioxidant responses during drought stress. These findings enhance our understanding of drought adaptation in rubber seedlings and provide valuable information for future studies on resilient genotypes, promoting sustainable rubber production and laying a foundation for further research on drought resilience in rubber trees and related species.

## Supporting information

S1 Data Data_PlosOne. (DOCX)
