# Supplementary material for: Physiological and gene expression analysis of Hevea brasiliensis under drought stress
Source: PLoS One. 2025 Dec 30;20(12):e0338177. doi: 10.1371/journal.pone.0338177 (PMC12752943; doi:10.1371/journal.pone.0338177)
Supplement: S1 Data — (DOCX) [file pone.0338177.s001.docx]

|  |  | Before stress | |  | After stress | |  | Difference |  |
| --- | --- | --- | --- | --- | --- | --- | --- | --- | --- |
| Treat | Rep | Height | Stem diameter |  | Height | Stem diameter |  | Height | Stem diameter |
| 1 | 1 | 42 | 3.94 |  | 43.5 | 4.3 |  | 1.5 | 0.36 |
| 1 | 2 | 42 | 3.36 |  | 43.5 | 3.54 |  | 1.5 | 0.18 |
| 1 | 3 | 45 | 3.6 |  | 48 | 3.76 |  | 3 | 0.16 |
| 1 | 4 | 37 | 4.3 |  | 41.5 | 4.6 |  | 4.5 | 0.3 |
| 1 | 5 | 39 | 3.24 |  | 42 | 3.44 |  | 3 | 0.2 |
| 2 | 1 | 43 | 4.64 |  | 45 | 4.68 |  | 2 | 0.04 |
| 2 | 2 | 41 | 2.84 |  | 41 | 2.85 |  | 0 | 0.01 |
| 2 | 3 | 41 | 3.94 |  | 41 | 4.18 |  | 0 | 0.24 |
| 2 | 4 | 40 | 3.42 |  | 40.5 | 3.52 |  | 0.5 | 0.1 |
| 2 | 5 | 43 | 3.46 |  | 43.5 | 3.51 |  | 0.5 | 0.05 |
| 3 | 1 | 48 | 4.8 |  | 48 | 4.94 |  | 0 | 0.14 |
| 3 | 2 | 42 | 5.3 |  | 43 | 5.5 |  | 1 | 0.2 |
| 3 | 3 | 42 | 2.85 |  | 42 | 2.96 |  | 0 | 0.11 |
| 3 | 4 | 41 | 2.74 |  | 41 | 2.9 |  | 0 | 0.16 |
| 3 | 5 | 40 | 2.7 |  | 40 | 2.86 |  | 0 | 0.16 |

|  | RWC | GPX | APX | CAT | MDA | PROLINE | HMGR | HMGS | RTA | CPT |
| --- | --- | --- | --- | --- | --- | --- | --- | --- | --- | --- |
| T11 | 91.09792 | 0.155 | 0.161 | 0.123 | 32.58 | 0.031 | 0.989041 | 0.9998997 | 1.011293 | 1.016362 |
| T12 | 95.11059 | 0.122 | 0.173 | 0.141 | 36.97 | 0.023 | 1.549155 | 1.012177 | 1.125731 | 1.2045 |
| T13 | 90.30612 | 0.217 | 0.203 | 0.177 | 50.52 | 0.025 | 0.461805 | 0.987924 | 0.862977 | 0.779137 |
| T21 | 70.40816 | 0.193 | 0.464 | 0.389 | 79.74 | 0.01 | 0.189465 | 1.059463 | 0.652176 | 1.427344 |
| T22 | 68.56634 | 0.271 | 0.403 | 0.442 | 120.39 | 0.008 | 0.268408 | 1.122465 | 1.065603 | 2.388045 |
| T23 | 67.59167 | 0.195 | 0.436 | 0.409 | 133.03 | 0.011 | 0.100481 | 0.96531 | 0.431272 | 0.959816 |
| T31 | 54.83625 | 0.357 | 0.285 | 0.147 | 202.71 | 0.051 | 0.055939 | 0.793701 | 0.672062 | 3.174802 |
| T32 | 55.54739 | 0.384 | 0.245 | 0.223 | 201.03 | 0.054 | 0.071669 | 0.87005 | 1.124411 | 4.03014 |
| T33 | 50.47096 | 0.32 | 0.194 | 0.229 | 249.87 | 0.066 | 0.04145 | 0.745701 | 0.426811 | 2.515474 |
